# The Multifaceted Effects of Omega-3 Polyunsaturated Fatty Acids on the Hallmarks of Cancer

**DOI:** 10.1155/2013/261247

**Published:** 2013-05-16

**Authors:** J. A. Stephenson, O. Al-Taan, A. Arshad, B. Morgan, M. S. Metcalfe, A. R. Dennison

**Affiliations:** ^1^Department of Cancer Studies and Molecular Medicine, University of Leicester, Leicester Royal Infirmary, Leicester LE1 5WW, UK; ^2^Department of Imaging, Leicester Royal Infirmary, Leicester LE1 5WW, UK; ^3^Department of Surgery, University Hospitals of Leicester, Leicester General Hospital, Leicester LE5 4PW, UK

## Abstract

Omega-3 polyunsaturated fatty acids, in particular eicosapentaenoic acid, and docosahexaenoic acid have been shown to have multiple beneficial antitumour actions that affect the essential alterations that dictate malignant growth. In this review we explore the putative mechanisms of action of omega-3 polyunsaturated fatty acid in cancer protection in relation to self-sufficiency in growth signals, insensitivity to growth-inhibitory signals, apoptosis, limitless replicative potential, sustained angiogenesis, and tissue invasion, and how these will hopefully translate from bench to bedside.

## 1. Introduction

Fatty acids (FAs) are a diverse group of molecules. The fatty acyl structure represents the major building block of complex lipids and FAs should be regarded as one of the most fundamental categories of biological lipids [1]. Fatty acids are key nutrients that affect early growth and development, as well as chronic disease in later life. The benefits and potential risks of FAs go well beyond their defined role as fuel [2].

An FA containing more than one carbon double bond is termed polyunsaturated fatty acid (PUFA). The most important families in human metabolism are omega-6 (n-6) and omega-3 (n-3) PUFAs. Specific n-6 and n-3 PUFAs are essential nutrients, while the eicosanoids and docosanoids they derive have distinct biological activities affecting the prevalence and severity of cardiovascular disease, diabetes, inflammation, cancer, and age-related functional decline [1, 2].

Important n-3 PUFAs involved in human nutrition are *α*-linolenic acid (ALA or 18 : 3n-3), eicosapentaenoic acid (EPA or 20 : 5n-3), docosapentaenoic acid (n-3 DPA or 20 : 5n-3), and docosahexaenoic acid (DHA or 22 : 6n-3).

ALA is the parent FA of the n-3 PUFA family. ALA is mainly found in the plant kingdom with high concentrations in flaxseed oil and perilla oil. It is also found in canola oil, soybean oil, and vegetable oils from where humans derive it in their diet. The human body is unable to readily synthesize ALA, which makes ALA, like linoleic acid (LA or 18 : 2n-6), the parent of the n-6 PUFA family, an essential fatty acid [1].

LA and ALA are converted to their respective n-6 and n-3 PUFA families by a series of independent reactions. However both pathways require the same enzymes for desaturation and elongation. This leads to competition between n-6 and n-3 PUFA for their metabolic conversion. The first step in the pathway requires Δ6 Desaturase [3, 4] which has a higher affinity for ALA than LA but due to the typically higher intake and concentration of LA there is greater conversion of n-6 PUFA producing the predominant product of the n-6 pathway, arachidonic acid (AA or 20 : 4n-6) [1, 5–7]. Thus the capacity of human metabolism to derive EPA and DHA by the desaturation of ALA is negligible in normal circumstances [1]. The efficiency of conversion is particularly poor in relation to DHA [6, 8]. The concentration of EPA and DHA in tissues can however be enhanced by direct ingestion of either oily fish or as a fish oil (FO) supplement or when competing amounts of n-6 PUFAs are relatively small [8–10].

Fish are able to build up large concentrations of n-3 PUFAs in their tissues by consuming algae and plankton and are therefore the main dietary source of essential n-3 PUFAs in humans. In particular cold-water oily fish such as mackerel, salmon, herring, anchovies, sardines, and smelt provide relatively large amounts of EPA and DHA [7].

## 2. Physiological Effects of Omega-6 and Omega-3 Polyunsaturated Fatty Acids

n-6 and n-3 PUFAs have a number of vital functions in the human body [11, 12]. As components of structural phospholipids in the cell membrane, they modulate cellular signaling, cellular interaction, and membrane fluidity [13].

They regulate the immune system by acting as precursors for eicosanoids-potent immunoregulatory metabolites. Eicosanoids are synthesised from the n-6 PUFA arachidonic acid (AA, 20 : 4n-6) and the n-3 PUFA, EPA. AA and EPA are metabolised by cyclooxygenase (COX) or lipoxygenase (LOX) enzymes into immunoregulatory metabolites prostaglandins (PGs), thromboxanes (TXs), and leukotrienes (LTs) [13]. As cell membrane phospholipids generally contain significantly higher levels of AA than EPA [14], AA is the most common eicosanoid precursor and gives rise to 2-series PGs and TXs and 4-series LTs. EPA gives rise to 3-series PGs and TXs, 5-series LTs, and E-series resolvins [13, 15].

DHA is a poor substrate for COX and LOX and it was thought that DHA did not produce bioactive COX and LOX mediators. However, Serhan and others identified bioactive docosanoids, named D-series resolvins and protectins [15–17].

AA and EPA also compete for the COX and LOX enzymes. Again, n-3 PUFAs are preferentially used, so supplementation with n-3 PUFAs will have a considerable impact on the production of eicosanoids and docosanoids. Thus, increased intake of n-3 PUFAs results in decreased generation of AA-derived eicosanoids and increased EPA derived eicosanoids and DHA docosanoids [18–21].

It is considered that the eicosanoids and docosanoids produced from EPA and DHA have less biological activity. Therefore have the advantage of being less pro-inflammatory in their action than the potent pro-inflammatory AA-derived mediators [13, 16, 22]. It is also suggested that they also have properties which are anti-inflammatory [15–17].

This theoretical benefit is the rationale for the use of FO supplements in chronic inflammatory disease such as asthma [23] and rheumatoid arthritis [22]. It is also why there is significant interest in the use of n-3 PUFA supplementation in critically ill patients and in patients undergoing major surgery [24–38].

## 3. The Role of Polyunsaturated Fatty Acids in Tumourigenesis

Hanahan and Weinberg in their landmark review “The hallmarks of cancer” and the subsequent “Hallmarks of the Cancer: the next generation” suggested that the vast catalog of cancer cell genotypes is a manifestation of essential alterations in cell physiology that collectively dictate malignant growth [39, 40].

The original six essential alterations described are self-sufficiency in growth signals, insensitivity to growth-inhibitory (antigrowth) signals, evasion of programmed cell death (apoptosis), limitless replicative potential, sustained angiogenesis, and tissue invasion and metastasis. This results in the cancerous cell having the predatory properties that allow it to survive, invade, and multiply where it should not. Recently the addition of reprogramming of energy metabolism and evading immune destruction has been suggested. Each of these physiologic changes (novel capabilities acquired during tumor development) represents the successful breaching of anticancer defense mechanisms. They proposed that these capabilities are shared in common by most and perhaps all types of human tumors and must be satisfied for tumour growth to occur within the tumour microenvironment [39, 40].

**Figure 1:**
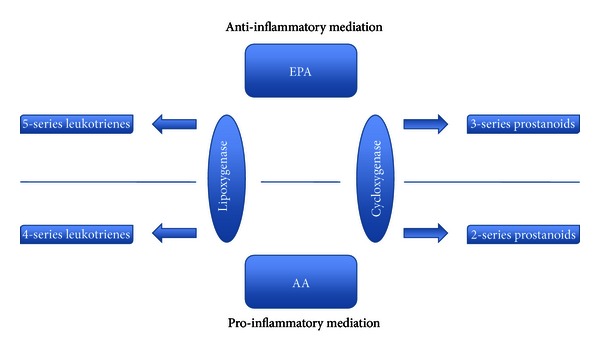
Inflammatory mediators derived from eicosapentaenoic acid and arachidonic acid. Adapted From Furst 2000.

EPA and DHA have been shown to have multiple anti-tumour actions that affect all of the original six essential alterations that dictate malignant growth. This is a result of various pathways including inhibition of AA metabolism and independent effects on various cytokines involved in tumourigenesis. n-6 PUFA derived eicosanoids have promoting effects in cancer cell growth [41, 42], angiogenesis [42], and invasion [43]. As previously discussed n-3 PUFAs can also be metabolized to resolvins and protectins [15, 44]. These compounds possess immunoregulatory actions [45] and it is well documented that inflammation plays an important role in the development of numerous human malignancies [46–48]. Thus one of the possible mechanisms for inhibition of tumor growth by n-3 PUFAs is via immunoregulation through production of 5 series leukotrienes (LT), 3 series prostaglandins (PG) and thromboxanes (TX), and resolvins—Figure 1 [49].

## 4. Effects of Omega-3 Polyunsaturated Fatty Acids on Growth Signals

Normal cells are unable to proliferate in the absence of stimulatory signals from transmembrane receptors, which are activated by growth factors, extracellular matrix components, and cell-cell interaction molecules [39]. Conversely, tumour cells however have a reduced dependence on such exogenous growth signals. Cancerous cells often bypass this step by synthesizing their own growth factors [50], overexpressing cell surface receptors which transmit growth-stimulatory signals [50, 51] or switching integrins to ones which favour growth signal transition [39–52]. Also many oncogenes mimic normal growth signals, promoting proliferation [39, 53].

The overall result is that the cancer cell is self sufficient in stimulating its own multiplication. The reduced dependence on exogenous growth signals and stimulation from normal tissue microenvironment leads to unregulated and exponential growth.

### 4.1. *In Vitro* Evidence

The cell plasma membrane affects growth factor: receptor interaction and subsequent signal transduction. EPA and DHA have been shown to have beneficial effects on the plasma membrane in MDA-MB-231 breast cancer cells with a marked decrease of epidermal growth factor receptor (EGFR) in lipid rafts, leading to alteration in EGFR signaling in a way that decreases the growth of breast tumors [54].

n-3 PUFAs appear to downregulate protein kinase C *β*2 [55, 56], RAS [57], and nuclear factor *κβ* (NF-kB) [58] which are important cell signaling mediators often found to be elevated in carcinogenesis. 

DHA has also been shown to modulate heat shock proteins that act as “chaperones” in protein: protein interactions and in cell membrane transport. [59]. DHA is also known to modulate steroid receptors in human cancer cell lines [60].

Tumour derived nitric oxide (NO) has the ability to promote tumour growth by enhancing invasiveness of tumour cells [61, 62]. NO also increases PGE2 production, which is implicated in tumour growth and progression [62]. EPA and DHA suppress NO production in macrophage cell lines in a dose dependant fashion [63, 64].

EPA and DHA inhibited human colon adenocarcinoma Caco-2 cell proliferation. Cells cultured with EPA or DHA reached much lower final densities compared to cells cultured with LA. The authors proposed that low insulin growth factor II (IGF-II)/IGF binding protein-6 ratios may have resulted in less free IGF-II a potent cell proliferation promoter and, consequently, the slower proliferation of Caco-2 cells treated with EPA or DHA [65].

### 4.2. *In Vivo* Evidence

COX-2 over-expression has been reported in 90% of colon tumours and colonic adenomas [66]. COX-2 has direct and indirect effects on growth via upregulation of growth signals and prostaglandins, angiogenesis, apoptosis, and cell-cell interaction [67]. The specific effects will be discussed in each subsequent section. In relation to self-sufficiency, numerous studies have found that COX-2 and its active metabolite PGE2-levels are reduced by supplementation of n-3 PUFAs [66, 68, 69]. A prostate cancer cell xenograft in mice found that the reduced levels of COX-2 and PGE2 were related to a reduction in tumour growth rate, tumour volume, and serum PSA [70].

Protein kinase C (PKC) Δ has a tumour suppressor function. The carcinogen azoxymethane decreases levels of PKC Δ. This decrease has been shown to be ameliorated in rats feed FO [71]. PKC *β*2, which is induced early in colon carcinogenesis, leading to self-sufficiency, cancer promotion, and carcinogen induced epithelial hyper-proliferation [72–76], is significantly decreased in rats fed FO. This blocked PKC *β*2 hyperproliferation [74, 77].

**Figure 2:**
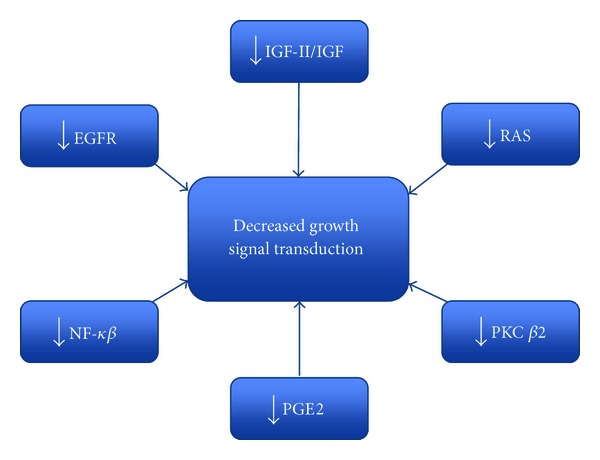
Multi-modal putative mechanisms of action of DHA and EPA on growth signal transduction.

The effect of n-3 PUFAs on growth signal transduction appears to be multi-faceted, with numerous putative pathways identified in the *in vitro* and *in vivo* setting (Figure 2). This suggests that any relationship of n-3 PUFA on tumourigenesis is complex.

## 5. Effects of Omega-3 Polyunsaturated Fatty Acids on Tumour Insensitivity to Growth-Inhibitory Signals

Tissue homeostasis and cellular quiescence is maintained in normal cells by anti-proliferative signals from growth inhibitory factors (tumour suppressor genes) and from cell-cell or cell-extracellular matrix interaction. These antigrowth signals are transmitted by cell surface receptors and may have 2 potential effects: (1) cells are forced into the quiescent (G0) state; (2) cells are induced into a post-mitotic state of permanent dormancy [39].

### 5.1. *In Vitro* Evidence

Investigation of the effect of EPA and DHA on colon cancer cell lines has shown decreases in cellular proliferation. Mengeaud demonstrated that cellular proliferation in HRT-18, HT-29, and Caco-2 cell lines is decreased by EPA [78]. This was replicated in a SIC oncogene transformant cell line by Tsai who also showed that DHA reduced cellular proliferation [79]. In two studies using HT-29 cell lines Clarke reported that EPA reduced cell proliferation and Chen demonstrated that DHA had a similar effect [80, 81]. Other studies have also shown decreases in cell proliferation in response to EPA and DHA [65, 82].

### 5.2. *In Vivo* Evidence

In a rodent model of breast cancer, DHA induced a reduction in mammary tumours accompanied by a 60% upregulation of BRCA1 tumour suppressor protein [83].

Numerous studies have shown a decrease in tumour cellular proliferation in response to n-3 PUFAs; however the putative mechanisms are not well documented and further investigation is required.

## 6. Effects of Omega-3 Polyunsaturated Fatty Acids on Tumour Evasion of Programmed Cell Death

Apoptosis governs the rate of cell attrition. The ability of tumour cells to expand in number is governed by the balance of proliferation and apoptosis. Evasion of apoptosis allows tumour cell mass to increase dramatically and it is a hallmark of tumourigenesis.

### 6.1. *In Vitro* Evidence

The studies previously reported on HT-29 colon cell lines by Clarke and Chen also showed increased induction of apoptosis with n-3 PUFAs [80, 81]. Other studies have shown that DHA induces a dose-dependant effect upon cancer cell apoptosis [84–86].

DHA has been shown to induce cytochrome c release, which binds to apoptotic protease activating factor initiating cancer cell apoptosis [85, 87].

n-3 PUFAs alter peroxisome proliferator-activated receptors (PPARs) cell signaling by acting as direct ligands for the receptors. DHA has been shown to modulate PPAR receptor expression [88, 89] and induce cellular apoptosis [67, 90, 91]. This was mediated through the effect of PPAR on Syndecan-1 a protein product, which induces apoptosis [67, 91].

EPA and DHA have also been shown to modulate expression of the Bcl-2 family. They downregulate the expression of anti-apoptotic proteins Bcl-2 and Bcl-xL and increase levels of Bak and Bcl-xS pro-apoptotic proteins [92–97].

NF*κβ*, which has the ability to block programmed cell death potentiating tumour survival, is downregulated by n-3 PUFAs in murine macrophages, which decreases COX-2 expression restoring functional apoptosis [23, 98].

### 6.2. *In Vivo* Evidence

Hong showed that in a mouse model of colon carcinogenesis that initiation of tumour growth was restricted by increased apoptosis related to n-3 PUFA supplementation [99]. One way in which apoptosis may be regulated by n-3 PUFAs is via COX-2. COX-2 has been shown to decrease apoptosis via expression of the Bcl-2 gene. A reduction of COX-2 and COX-2 inhibition have been shown to repress the expression of Bcl-2 gene and its anti-apoptotic properties [67, 67, 69, 91].

The Bcl-2 family also has a pro-apoptotic member Bad. In its normal state Bad promotes cell death by displacing Bax from Bcl-2 [100, 101]. Phosphorylation of Bad prevents it from displacing Bax from Bcl-2 subsequently promoting cell survival [100, 102, 103]. A study by Berquin on Pten knockout mice showed that prostate tumours from mice with an enriched n-3 PUFA diet had lower levels of phosphorylated Bad and higher apoptotic indexes compared to mice on an n-6 PUFA diet. This led to reduced tumour growth, slowed histopathological progression, and increased survival rates [49].

Tumour evasion of programmed cell death is a complex and controlled by an intricate milieu of intra-cellular signal transduction pathways and external cytokines, survival factors, chemokines, growth factors, and death factors. Evidence suggests that DHA and EPA have effects on many of these pathways, which seem to be beneficial.

## 7. Effects of Omega-3 Polyunsaturated Fatty Acids on Limitless Replicative Potential of Tumours

Growth signal autonomy, insensitivity to antigrowth signals, and apoptotic evasion alone do not lead to expansive tumour growth because cells have the capacity for senescence, an intrinsic property that limits multiplication [39, 104]. Senescence can be circumvented by DNA damage and disabling tumour suppressor genes such as p53 and pRb, which eventually leads to immortalisation or the ability to multiply without limit [105].

### 7.1. *In Vitro* Evidence

AA may promote tumour growth and replication via activation of protein kinase C stimulating mitosis [106]. Studies in colonocytes and JB6 cells—mouse epidermal cells—have shown that growth promotion via the transcription factors RAS and AP1 is reduced by n-3 PUFAs [107, 108]. The second messengers of AA metabolism with COX and LOX also stimulate mitosis. Conversely EPA derived metabolites of COX and LOX have been shown to decrease growth of human breast cancer cell lines [42].

### 7.2. *In Vivo* Evidence

Numerous animal study models in colon cancer have demonstrated that n-3 PUFA supplementation leads to tumour growth suppression [68, 109–115].

It had been demonstrated in the rat colon that n-3 PUFAs reduced k-RAS mutations and decreased membrane RAS expression [116] and it has been suggested that this indicates that n-3 PUFAs may protect against colon carcinogenesis by decreasing DNA adduct formation and/or enhancing DNA repair [117]. In the study already discussed by Hong they also showed that there was a reduction in DNA adduct formation [99]. Reddy showed that initiation of experimentally induced colon cancer was reduced by the protective effect of n-3 PUFAs [118].

In xenografted rats carrying neuroblastoma tumours, Gleissman demonstrated that DHA-enriched diet prior to tumour cell injection delayed tumour formation and prevented tumour establishment [119]. In the same study Gleissman investigated the effect of DHA as a therapeutic agent in rats who had established tumours. Tumours in animals receiving high dose DHA showed partial response compared to animals receiving low dose DHA or control that showed stable disease and progressive disease, respectively [119].

Another therapeutic study performed in nude mice xenografted with BxPC-3 pancreatic cancer cells showed tumour inhibition by DHA. Interestingly the inhibition was increased in another group where DHA was combined with curcumin [120].

## 8. Effects of Omega-3 Polyunsaturated Fatty Acids on Sustained Angiogenesis

For a tumour to grow beyond 2 mm, angiogenesis and neovascular formation are required. The ability to induce and sustain angiogenesis from vascular quiescence is controlled by the “angiogenic switch.” Tumors appear to activate the angiogenic switch by changing the balance of angiogenesis inducers and countervailing inhibitors [121]. This is seen with increased production, expression, and signal transduction of pro-angiogenic factors such as vascular endothelial growth factor (VEGF). n-3 PUFAs have been shown to have a profound effect on angiogenesis [122].

### 8.1. *In Vitro* Evidence

n-3 PUFAs have been shown to decrease sprouting angiogenesis by suppressing VEGF-stimulated endothelial cell proliferation, migration, and tube formation [123–125]. Tsuzuki and colleagues treated human umbilical vein endothelial cells with conjugated EPA and demonstrated that a reduction in sprouting angiogenesis tube formation and endothelial cell migration [123] was also seen in bovine aortic endothelial cells pre-treated with DPA. VEGF-Receptor (VEGF-R) 2 expression was also found to be suppressed [124]. The reduction in endothelial cell proliferation in response to EPA was shown to be dose dependant in bovine carotid artery endothelial cells [125]. The study by Yang et al. also elicited a dose dependant decrease in VEGF-1 (FlK-1) expression [125]. A reduction in VEGF/VEGF-R binding has also been demonstrated by Yuan et al. using an n-3 PUFA rich shark oil [126].

n-3 PUFAs also have stark effects on numerous other mediators involved in angiogenesis. Platelet derived growth factors (PDGF) play an important role in angiogenesis by stimulating fibroblast and vascular smooth muscle cell motility and acting as a chemo-attractant [127, 128]. As early as 1988 Fox and DiCorleto showed that *in vitro* production of PDGF was inhibited by n-3 PUFAs [129]. Investigating the effects of EPA and DHA on PDGF signal transduction Terano and colleagues demonstrated that EPA inhibited PDGF binding to its receptor and suppressed c-fos mRNA expression, a gene involved in receptor signal transduction. These effects led to inhibition of smooth muscle proliferation a prerequisite for angiogenesis [130].

As previously discussed PGE2 is formed from AA, catalysed by COX-2. There is well-defined link between E series prostaglandins and carcinogenesis [131]. Decreased levels of VEGF, COX-2, and PGE2 have been demonstrated in HT-29 colon cancer cell lines when cultured *in vitro* with EPA and DHA [112] and a synergistic inhibitory effect of n-3 PUFAs and COX-2 inhibitors on growth of human colon cancer cell lines has been shown [60, 132].

Nitric oxide (NO) promotes endothelial cell survival and proliferation and inhibits apoptosis [133, 134]. NO and COX-2 also regulate VEGF-mediated angiogenesis [135–137]. Inducible nitric oxide synthase (iNOS) stimulates NO production [136].

DHA has been demonstrated to inhibit NO production and iNOS expression in murine macrophages [63, 64, 138–140] and downregulate NO and nuclear factor kappa beta (NFKB) in human colon cancer cell lines [141].

In the study previously discussed by Tsuzuki et al., they also demonstrated that production of matrix metalloproteinases (MMP) 2 and 9—proteases which play a role in basement membrane proteolysis in the 3rd stage of sprouting angiogenesis—in human endothelial cells was inhibited by EPA [123].

It has also been demonstrated that DHA inhibits Beta-catenin—a transcriptional regulator of angiogenesis—production in colon cancer cells [142].

### 8.2. *In Vivo* Evidence

In a study where Fischer 344 rats were implanted with fibrosarcomas, the group with diets supplemented with EPA had tumours with significantly lower tumour volume and decreased VEGF-alpha mRNA levels [143].

A study in nude mice supplemented with n-3 PUFA undergoing implantation of human colorectal carcinomas showed that tumour expression of VEGF, COX-2, and PGE2 was decreased compared to control [112]. Benefits were also seen in nude mice transplanted with breast carcinoma. Breast tumours in mice feed diets high in EPA and DHA had lower tumour microvessel density and VEGF levels compared to controls [144, 145].

Induction of vascular smooth muscle cell migration by PDGF, required for angiogenesis, is inhibited by EPA and DHA *in vivo* [130]. Several other small animal models have demonstrated that n-3 PUFA enriched diets inhibit COX-2 and PGE2 production [146] and reduced HT-29 colon cancer cell tumour growth and microvessel density after implantation into nude mice [112].

**Figure 3:**
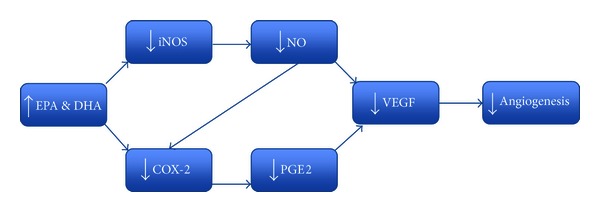
Pathways leading to the anti-angiogeneis effect of DHA and EPA.

Factors such as PGE2, NO, COX-2, and NFKB have well-documented roles in both the inflammatory and angiogenic cascades with significant cross-relation in both pathways. This demonstrates the potential for n-3 FAs as anti-angiogenic agents via inhibition of these factors and others including VEGF and PDGF (Figure 3).

## 9. Effects of Omega-3 Polyunsaturated Fatty Acids on Tumour Tissue Invasion and Metastasis

Metastases are the cause of 90% of human cancer related deaths [147]. Like the formation of the initial tumour the above 5 characteristics are required for metastasis formation. Metastasis and tissue invasion also require loss of cell-cell adhesion-regulated by cell adhesion molecules (CAMs)—and cell-ECM interactions-regulated by integrins [39, 148]. E-cadherin, a CAM, is lost in the majority of epithelial cancers, which enables invasion and metastases [149]. Once at a new site tumour cells then shift the expression of integrins to facilitate preferential cell binding to allow the tumour to “seed” leading to subsequent distant growth. 

### 9.1. *In Vitro* Evidence

DHA has been shown to reduce the induction of monocyte rolling, adhesion, and transmigration controlled by TNF*α* [150].

As previously discussed NO increases tumour growth and angiogenesis [61, 62, 151–153]. NO also plays an important role in tumour cell migration, which may be decreased by EPA and DHA supplementation due to suppression of tumour derived NO production [63, 64].

Cell-cell adhesion is modulated by DHA via down regulation of Rho GTPase, which inhibits cytoskeleton reorganisation [154], and reduction in ICAM-1 and VCAM-1 expression [155]. 

### 9.2. *In Vivo* Evidence

Again the COX-2 pathway plays an important role in each of the tumour development pathways. COX-2 reduces cell-cell and cell-matrix interactions leading to increased progression and metastases of gastric carcinoma [156, 157]. COX-2 inhibition has been shown to reduce invasiveness and depress metastases of gastric cancer in various animal models [67]. A xenograft animal model showed inhibition of tumour cell growth and invasion by n-3 PUFAs associated with decreased COX-2 and PGE2 levels [70]. n-3 PUFAs may act as a natural COX inhibitor [158]. Despite the wealth of evidence of the beneficial anti-tumour effects of DHA and EPA via downregulation of the COX-2 pathway a study by Boudreau has suggested that there are also COX-2 independent methods of protective action. In a colon cancer xenograft model, tumour formation was inhibited by n-3 PUFA supplementation in both COX-2 deficient and COX-2 overexpressing tumours [111]. 

Inhibition of metastases by n-3 PUFA enriched diets was demonstrated in both mouse and rat [159, 160] models of colorectal cancer. EPA and DHA have also been shown to suppress development of lung metastases due to reduced 72-kDa type IV collagenase gelatinolytic activity [161].

There is a large and growing body of evidence from laboratory-based studies that n-3 PUFAs have a marked beneficial effect on the hallmarks of cancer. However do these mechanistic studies translate into a clinical benefit?

## 10. Effects of Omega-3 Polyunsaturated Fatty Acids on Tumourigenesis in Humans

As well as the cellular mechanisms described in the *in*-*vitro* and *in*-*vivo* studies above, epidaemiological observations also appeared to suggest a benefit of n-3 PUFA in cancer prevention in humans. An example is an observational study in Inuits—Inuits have DHA levels several times higher than Caucasians [162]—which has demonstrated significantly lower levels of childhood cancer occurrence, particularly neuroblastoma—tenfold decrease—and Hodgkin lymphoma [92, 163].

However, a systematic review of the “Effects of omega-3 fatty acids on cancer risk” by MacLean et al. reviewed 38 articles published from 1966 to 2005 which included 65 estimates of association calculated over 20 differing cohorts for 11 different cancer types concluded that only 10 were statistically significant and that the body of literature does not provide evidence to suggest a significant association between n-3 PUFA and cancer incidence. They also stated that dietary supplementation with n-3 PUFAs is unlikely to prevent cancer [164]. Chen et al. raised concerns with the systematic review [165]. The studies included in this systematic review did not formally measure FA consumption-food frequency questionnaires and dietary records were used which correlate poorly with direct PUFA measurement [166]—The studies included in this systematic review did not formally measure FA consumption, they used food frequency questionnaires and dietary records, which correlate poorly with direct PUFA measurement [166] and do not differentiate between the source of FO consumption [165].

Animal data—some of which has been discussed above—is an invaluable tool for mechanistic studies and the models can closely mimic the clinical course of cancer progressions [165, 167, 168]. However the translation of animal data into the clinical arena is difficult due to the higher amounts of n-3 PUFAs used in relation to fat intake and percentage weight [164]. Inherent to the majority of animal studies is the use of high levels of dietary constituents [117] with n-3 PUFA intake between 18 and 48% of daily energy compared to 4–10% in human population based studies [169]. This is likely to be one of the reasons that only weak associations of PUFA intake and cancer are found in population-based studies [117]. Extrapolation of findings is also confounded by poor descriptions of experimental conditions and dose and purity of n-3 PUFA supplementation [164, 170].

However, a role is potentially being developed for n-3 PUFA in combination with current chemotherapeutic agents to augment their action [171]. Animal models have shown that the efficiency of doxorubicin [172] and mitomycin C [173] in inhibiting tumor growth and the inhibitory effect of tamoxifen in estrogen-dependent xenografts [174] are enhanced when combined with n-3 PUFA-enriched diets. 

DHA supplementation with concurrent cytotoxic drug treatment is potentially a way in which to clinically utilise DHA in cancer treatment. DHA in combination with doxorubicin, irinotecan, cisplatin, melphalan, and vincristine on neuroblastoma cell survival shows additive or synergistic interactions [85, 92].

A therapeutic study in breast cancer patients where DHA was combined with the chemotherapeutic drugs epirubicin, cyclophosphamide, and 5-fluorouracil showed delayed time to tumour progression and longer overall survival. However these findings were only observed when patients were stratified into 2 groups of either high or low incorporation of DHA into plasma and erythrocytes. Patients who had high incorporation of DHA into plasma and erythrocytes benefitted compared to those who had low level DHA incorporation [175]. This observation correlates well with other studies showing that DHA incorporation differs between individuals due to dissimilar rates of metabolism, enzymatic activity, background diet, age, and sex [92, 176–178]. This is likely to be a recurrent problem in studies using oral n-3 PUFA supplementation.

## 11. Discussion

In the last decade there has been a growing interest in the role of FAs, especially PUFAs, in cancer development and progression. As discussed the link between FAs and cancer may relate to the synthesis of eicosanoids, which have wide-ranging diverse effects at a cellular level. There are currently several ongoing clinical trials to assess this, where n-3 PUFAs are being tested for cancer prevention, support, or therapy [158], but initial evidence suggests that researchers do not seem to be translating the profoundly beneficial results seen in the laboratory to the bedside. This is potentially due to the way in which n-3 PUFAs are being supplemented and we need to think about novel ways of overcoming the difficulties faced with FO supplementation to assess the true benefit of n-3 PUFAs in the fight against cancer. Maybe we also need to explore the broader therapeutic benefits of FO supplementation on areas such as cancer cachexia and aiding treatment tolerance as recently suggested by Murphy et al. [179]. However, ongoing and future clinical trials using intravenous n-3 PUFA infusions in cancer therapy are eagerly awaited.
